# A Constructivist Intervention Program for the Improvement of Mathematical Performance Based on Empiric Developmental Results (PEIM)

**DOI:** 10.3389/fpsyg.2020.582805

**Published:** 2021-01-13

**Authors:** Vicente Bermejo, Pilar Ester, Isabel Morales

**Affiliations:** ^1^Universidad Complutense de Madrid, Madrid, Spain; ^2^Facultad de Educación, Universidad Camilo José Cela, Madrid, Spain

**Keywords:** PEIM, constructivism, developmental dimension, problem solving, mathematics teaching

## Abstract

Teaching mathematics and improving mathematics competence are pending subjects within our educational system. The PEIM (*Programa Evolutivo Instruccional para Matemáticas*), a constructivist intervention program for the improvement of mathematical performance, affects the different agents involved in math learning, guaranteeing a significant improvement in students’ performance. The program is based on the following pillars: (a) students become the main agents of their learning by constructing their own knowledge; (b) the teacher must be the guide to facilitate and guarantee such a construction by being a great connoisseur of the fundamental aspects of the development of the child’s mathematical thinking; (c) the mathematical contents must be sequenced in terms of the complexity and significance for the student as well as contextualized at all times; and (d) the classroom must have a constructivist climate highlighting cooperative work among students. The implementation of PEIM along with the empirical evaluation conducted in several centers in Madrid and Zaragoza (Spain) confirm how students improve their mathematical competence. Both first- and second-grade students in elementary education were far more effective in solving problems, highlighting the use of more advanced strategies in their resolution and a lower incidence of conceptual errors. Moreover, it was possible to verify how the students proving greater difficulty, experienced an evolution in learning similarly to those who did not present it. The program provides customized education to allow the teacher to know at all times how he should be more influential on the students’ learning through mathematical profiles. Both teaching practice and teachers were observed, being that of the experimental group more prone to analyzing processes and allowing the construction of knowledge by students, due to their psycho-developmental training. As a result, we found several improvements through the implementation of the program that may serve, for upcoming years, as a basis for the necessary changes in the teaching of mathematics.

## Introduction

The emergence of research papers on the teaching-learning of mathematics is increasingly noticeable. The improvement of students’ training is beginning to be a matter of state due to the high levels of school failure in international evaluations. Research results are frequently a long way from the classroom reality. In other words, it seems that research and educational practice are walking along different paths, and as a result, bridges should be built between the university and the school (Bermejo, 2018). However, improving mathematical competence does not imply making faster calculations, or obtaining a better resolution in activities only when they are presented to students more or less regularly. Instead, it implies that our students learn in a meaningful way, building their own knowledge to permit them directly apply what they have learned in their daily life, and always starting from their previous knowledge.

It is unacceptable the mathematical schizophrenia that comes out and is materialized in the child when the informal knowledge runs in parallel with the instruction received in the classroom. For many authors, such as Russell and Ginsburg (1984), those learnings the child obtains on a daily basis, and therefore, all students possess since they are first schooled, are the starting points to scaffold more precise and abstract concepts that will provide the learner with its straightforward application of what he has learnt in his upmost immediate environment.

On many occasions we can hear teachers speaking about the importance of calculus and the procedural mastery of arithmetic operations, relegating those activities that favor reasoning and problem solving.

We believe that it is important to disseminate research-based practices in which empirically contrasted positive results have been obtained, thus being able to obtain a number of evidence-based practices that allow improving the teaching-learning process and, therefore, the performance of students regarding mathematical competence. Educational practices that are research-validated can constitute a frame of reference to serve as a guide for knowledge transfer (Simplicio et al., 2020).

## Constructivism

The term “constructivism” comes from an artistic current that took place in present-day Russia around the year 1920 in the field of architecture and the plastic arts. However, the roots of this approach, as an epistemological proposal, go back even further than Plato with his innate ideas to justify knowledge. In fact, Gorgias (−380 BC) and the Greek sophists defended that we cannot know reality, but only have an opinion (“doxa”) on things. More recently, Descartes’ methodical doubt maintains that the only possible certainty is that of “cogito ergo sum.” Berkeley (1685–1753) proposes his well-known “esse est percipi,” to support that we only know our own ideas. In the same line, Giambattista Vico (1668–1744) affirms that man only understands what he does or builds. Likewise, Leibnitz’s well-known quote (1646–1716) follows the same direction: “nihil est in intellectu quod prius non-fuerit in senso, nisi ipse intellectus.” And finally, Kant (1724–1804) maintains that the mind is active and outlines experience (see Bermejo and Nieto, 2012).

Nevertheless, the father of cognitive constructivism is Jean Piaget with his work “La construction du reel chez l’enfant” published in Piaget (1937/1967), the second book of his well-known trilogy. As he concludes, accommodation and assimilation differ throughout the development of the child until they become increasingly complementary to each other: “True experience and deductive construction thus become both distinct and correlative” (p. 338). Therefore, constructivism takes up a room between empiricism and innatism or preformism, in an attempt to understand development as the result of an internal dimension formed by schemes, and another interactional dimension constituted by adaptation, being the result of the two above-mentioned functions: assimilation and accommodation. The former will help the subject adapt reality to his mental structures, whereas the latter will allow the adaptation of structures to reality. The equilibration of this process (equilibrium-disequilibrium-equilibrium) would be the fundamental cause of development, while factors such as social environment, physical environment and maturation become to play the facilitator roles (Piaget, 1937/1967). In this respect, the environment is not directly causing any development, but it can disturb or product disequilibrium. Therefore, equilibration, through a constructive process, would allow development to progress. However, equilibration is not understood as a static process, but a dynamic one, as Fosnot C. T. (1996) and Fosnot C. (1996b) highlights: “Equilibration is not a sequential process of assimilation, then conflict, then accommodation. Instead, it is a dynamic ‘dance’ of progressive equilibria, adaptation and organization, growth and change” (p. 14).

If the empiric learner is passive and relies upon effort and external motivation, and the maturing learner depends on an innate biological programming, the constructivist learner will evolve and develop via changes, equilibria and active constructions.

With some degree of frequency, different constructivist approaches are presented. Bermejo and Nieto (2012) talk about cognitive constructivism (Piaget), socio-cognitive constructivism (Vygotsky), biological constructivism (Maturana) and radical constructivism (Von Glaserfeld). Similarly, Castillo (2008) also proposes these four types of constructivism, although he classifies Maturana as a radical constructivist, along with Von Glaserfel. Nonetheless, in an attempt to analyze and coordinate them to pursue the main objective of the present paper, that is teaching-learning in the mathematics classroom, the following proposal presented by Cobb, seems to be accurate for us: “As was the case with the discussion of Rogoff’s and von Glasersfeld’s analyzes, this coordination of perspectives leads to the view that learning is both a process of self-organization and a process of enculturation that occurs while participating in cultural practices, frequently while interacting with others” (Cobb, 1996, p. 45).

In a few words, for Piaget “you only learn what you understand” and you only understand what you invent. This reminds us of the ideas of Giambattista Vico mentioned above. In contrast, Vygotsky refers to two levels of development (current and potential) and the zone of proximal development, highlighting the adult’s intervention in learning.

## Constructivist Intervention Programs

Teaching mathematics have been approached from many different disciplines, i.e., cognitive psychology, neuroscience, biology, genetics, etc., although some, such as mathematics teaching, science teaching and educational psychology construct closer bridges between their results and daily practice in the classroom. It must be taken into account that each of the different disciplines focuses on different variables of the teaching-learning process, the student, the context, the teacher, etc. Since the teaching process is very complex, the intervention programs must try to respond to all the variables involved in the process, being aware that modifications may occur while being implemented in the classroom, due to the introduction of variables by the agents involved, the latter being the bases that will allow carrying out new studies (Simplicio et al., 2020).

According to Cobb (1998), there are two fundamental reasons why constructivism can be an alternative to more traditional methodologies. The first reason considers that students are capable of solving a wide variety of mathematical problems because they develop more complex and abstract structures. And, the second reason, through the construction of their own knowledge, looks at students changing their perspective because they are capable of creating and controlling mathematics, thus increasing students’ motivation.

If we focus on learning mathematics, the interplay that occurs between the two approaches is the one that would allow the learning process to be balanced, since, as Bermejo et al. (2002) explains, the sociocultural part will focus on teacher-student and student-student interactions, and the participation of the individual to explain how the students take control of the teacher’s contributions. Cognitive theorists, however, would analyze the student’s processes of adaptation to the actions of others and would be more concerned with how deeply the individual interpretation is carried out. This implies that the construction of the individual mathematical concepts is influenced by the person’s interpretations of others’ activities and by his/her own.

In order to improve learners’ mathematical performance, our intervention program known as PEIM aims to improve the understanding of mathematical contents, specifically in problem solving tasks. In order to do so, it will directly apply to four parameters: students, teachers, curricular contents and the social climate of the classroom.

With respect to students, the program assumes constructivist approaches so as not to receive passive mathematical knowledge, but rather, to construct it by themselves. Nevertheless, it is necessary to consider the knowledge prior to learning, since the student comes to the classroom with the knowledge that they have been acquiring within their context based on their daily life, as proposed by Resnick (1992). Since the child is born, regardless of his cultural background, he grows in a context with multiple stimuli that influence his mathematical learning (Ginsburg and Seo, 1999). For this reason, it is essential for the child to integrate what he is proposed to do in class together with his previous knowledge, which would entail more meaningful learning and, at the same time, would make him become a more active subject in the classroom. Assuming this premise, it would allow us to avoid any rote and decontextualized learning and move away from more directive teachings in which processes take on a special role. When children find themselves in new situations, they must adapt to them by restructuring the surrounding context so that they can negotiate it more easily. And, usually, it is necessary that they use creativity and apply alternative or unconventional thinking to those situations (Bagassi et al., 2020). By means of the implementation of a constructivist intervention, the student constructs significant learning allowing him to be more mathematically competent on a daily basis. In this respect, a longitudinal study conducted in England shows how students, who studied using very different approaches, despite having similar teachers and curriculum, learned differently, and obtained attitudes toward mathematics also differently (Boaler, 2002a,b, 2015).

When we refer to learning as a constructive process, not all authors attribute the same meaning to these words (see Bermejo et al., 2000b). Lampert (1989), for example, maintains that knowledge is constructed by the student in the same way as knowledge is constructed in the discipline of mathematics. Meanwhile, Carpenter et al. (1996, 2014) considers the process as an idiosyncratic construction by the student. And the NTCM (National Council of Teachers of Mathematics) proposes that “students should learn mathematics by comprehending, actively building new knowledge from experience and from their previous knowledge” (2000, p. 20).

From our point of view, and according to the National Council of Teachers of Mathematics (2000), an appropriate constructivist intervention would allow the student to interact with both the teacher and the rest of the students, using at all times different means to reason, relate, solve problems and communicate. It would also allow the student to anticipate and make conjectures of solutions based on mathematical arguments that validate what is stated. Furthermore, it will aid the learner to focus on solving problems that allow examples and counterexamples to be explored. Considering these aspects, it concludes by saying that the student, through reasoning, is capable of establishing conjectures and solutions, a process that will combine their prior knowledge with the concepts that they work collaboratively in the classroom by creating new knowledge structures.

The idea that we propose in the PEIM is that the teacher, through individual interviews, can define the student’s mathematical profile in order to find out what developmental stage the student is at, what informal knowledge has been acquired and what type of proposals will allow the learner to progress adequately according to his rate of development.

Concerning teachers, they are a fundamental pillar in the teaching-learning process. It is essential for them to receive an extensive psycho-pedagogical training that enables them to get to know their students, so that they cannot only understand the mathematical content in a deeper way, but also to be aware of how each student learns mathematics, that is, to anticipate the child’s activity, their potential strategies and the mistakes committed in acquiring each content. It is, therefore, worth mentioning the importance that errors acquire as a source of learning. The understanding of the main mathematical concepts by the teachers will help them propose challenges to the student so that he/she can through meaningful learning, and construct more complex and abstract schemes that allow them to further develop their mathematical competence. Hence, it is important “to generate learning environments in which it makes sense both the approach and resolution of problems involving great mathematical ideas and those of other disciplines, and also the rules of the game used to deal with them” (Albarracín et al., 2018, p. 15).

From this perspective, it is essential that early childhood education teachers are aware of the most recent research findings in order to build bridges between research and classrooms, accommodating their own teaching to proven methods. With this respect, Koponen et al. (2016) proposes three priority areas in the development of the mathematical knowledge necessary for its teaching. The first area is concerned with a more coherent, comprehensive and shared understanding of what mathematics is, and how it should be taught. The second area calls for innovation and reflection on the research method. And finally, the third area involves carrying out studies on teaching, and deals with the nature of mathematical knowledge for a more equitable teaching.

For its part, the National Council of Teachers of Mathematics (2000) suggests taking into account several principles for teachers to design effective mathematics teaching: (a) to propose useful tasks that let them apply their knowledge to their daily life; (b) to analyze both the role of the teacher and the student in the educational practice, by using instruments that allow them to establish mathematical discussions to deepen their knowledge; and (c) to provide an adequate context previously analyzed adapting the teaching-learning process.

Cobb (1988, 1995) focuses on the idea that students construct their knowledge by restructuring their cognitive schemas and proposes that the teacher’s task does not longer consist in helping to receive and acquire mathematical knowledge, but to organize and structure the activities to be carried out by the student. In this way, the teacher’s role has substantially changed from the model presented by the traditional school. The teacher is no longer the instructor and becomes a guide to help students find their own way to solve the different activities proposed. This implies presenting situations where the student can look at different resolution strategies, making them do critical analyses and being able to justify how and why they did it in that way. For example, as Groen and Resnick (1977) put it, children are able to invent their own addition methods in the absence of adult instruction. This must let us think what, how and how much instruction is provided. Any connections between concepts as well as any applications provide a solid foundation for learning mathematics.

Likewise, the teacher’s mathematical language is modified to model the explanations of the students in more adequate terms, distancing themselves from models in which the appropriate answers are rewarded and mistakes are corrected.

As Jacobson (2017) states, teachers have to face many challenges in the classroom, and these allow them to move forward in developing the contents and methodologies. In this respect, however, we should not forget how necessary ongoing training is, since it involves accepting the two principles of constructivism, those that must be implicit and present in the classroom. On one hand, children construct their own knowledge and, on the other, teaching must be organized to facilitate and guarantee such knowledge construction in the most efficient way possible. Therefore, this training must be a priority issue in educational centers and holding seminars with a certain frequency will help improve teachers’ attitudes toward mathematics, and subsequently influence the instructional process in a positive way. Carpenter et al. (1998) show the existence of a close relationship between the change of beliefs in teachers and the way of teaching, as well as the performance of students (Yurekli et al., 2020). Furthermore, as Valentine and Bolyard (2019) write: “Negative attitudes toward mathematics are common among the general adult population, including prospective elementary teachers” (p. 437). So is this Philipp (2007), and the same belief is shared by students in terms of mathematics (Bermejo et al., 2000a). As some researchers have shown, there is a consistent relationship between math anxiety and performance (a medium to weak range, from −0.11 to −0.36), and they observe that those students who show higher in levels of MA tend to show poorer mathematics performance (Primi et al., 2020). None of these aspects are facilitators in the teaching-learning process.

We can then affirm that the learning process becomes a communicative process in which students are listened to, thereby understanding their goals and assuming their logical reasoning. The teacher in his role as a guide must ask students to clarify and justify their ideas both orally and in writing, which will allow him to influence and deepen into those aspects that he gauges more relevant, deciding when to relate language to mathematical notation. In order to do so, he will have to raise questions and provide assignments, provoke, compromise and challenge each student’s mind. “In order for the teacher to manage the discussion in class correctly and efficiently, he needs have not only extensive knowledge on the topic being discussed, but also, and above all, he needs to know how the child learns that specific topic, that is, the levels of development in understanding the topic or content, the difficulties and typical errors that usually arise” (Bermejo et al., 2002, p. 40). Hence the weight of the program falls on the teacher’s work before entering the classroom, because this, among other variables, will guarantee its success.

Another basic pillar of the program is school content. Quite a few studies show that the time spent on the subject, the parents and the content set by the government determine the educational practice (Anderson et al., 2005; Cross Francis, 2015; Yurekli et al., 2020). Therefore, it is necessary for the contents to be appropriately selected and sequenced. Such a selection does not largely affect the curriculum designed by the authorities, but what each teacher instructs on a daily basis. The selected activities should be aimed at facilitating comprehension, reasoning, solving verbal problems, preparing mental representations, making decisions, etc., and devoting less time to routine and mechanical activities. The contents that are worked on must be meaningful for the students and connected to their daily life. Bermejo et al. (2002) states that what is really important is that learning takes place in a real context for students, since it fosters self-confidence and makes their learning more significant. The sequencing of contents must be up to their difficulty criteria (Bermejo et al., 1998). In this way, the PEIM proposes micro-genetic studies that allow the teacher to know the child’s developmental steps regarding how each mathematical content is learned. In other words, it definitely suggests personalizing teaching.

Finally, the classroom context is another basic pillar making up the PEIM. In this respect, we understand that the context is the class dynamics and the multiple range of factors that facilitate and make learning possible. Some authors (see Saxe, 1991; Saxe and Guberman, 1998) understand learning as the individual achievement of goals through the development of collective activities. For this reason, Hatano and Inagaki (1991) believe that raising discussion among students would offer good opportunities for the construction of knowledge, due to socio-motivational factors. Likewise, cooperative work, in general, seems to have positive effects on learning. Cooperative learning helps students adopt different roles, from tutoring to being tutorized, and vice versa (Youde, 2020). Numerous studies (Johnson et al., 1983; Slavin, 1983a,b; Kagan, 1988; Johnson and Johnson, 1989; Sharan, 1990; Nelson-Le Gall, 1995) affirm that students who work cooperatively during their learning process, obtain global benefits at three levels: academic, social and personal. These benefits affect all students equally, from those whose profile is close to a proficient student to those who have learning difficulties (Huber and Carter, 2019; Moliner and Alegre, 2020; Sarid et al., 2020).

Authors such as Webb and Weeb and Farivar (1994) have studied this methodology in solving mathematical problems, although it must be said that it turns out difficult to identify the learning factors that influence on both the cognitive functions and the emotional sphere. Concerning these aspects, some authors conclude that peer interaction improves learning, because quite often does the child know his peers’ difficulties better than does the teacher himself. Cooperation leads them to share the way they think, acting as mediators in the way others think (Presseisen, 1992). For their part, Palincsar and Brown (1998) affirm that the dialogue between students leads them to understand the strategic aspects of learning, appreciating their own thoughts as tools to address problems, therefore, through such a dynamic exchange, they learn powerful dimensions of thought.

## The Present Study

The general objective of this study is to show the efficiency of the PEIM in learning mathematics, and, more specifically, in solving elementary verbal problems that require a single operation, either addition or subtraction, in the first years of elementary education. It is important to empirically demonstrate how likely it is to streamline the teaching-learning process by making methodological changes led by constructivist principles so as to improve students’ mathematical performance. We want to emphasize the importance of the developmental dimension of the program that will facilitate the teacher to be aware of the constructive process the child follows in the acquisition of new mathematical contents.

There are two fundamental reasons why a constructivist perspective can be an alternative to transmission-based teaching methodologies. On one hand, it allows students to create more complex and abstract strategies, thus strengthening the ability to solve problems in a significant way. And on the other, it provides students with a sense of control that motivates and makes them be conscious that they are capable of learning and construct mathematics through problem solving.

This study presents two empirical works that were implemented in two discrete autonomous communities in Spain. The first study took place in public state schools in the community of Madrid, and the second in charter schools in the community of Aragon. The first study was carried out in the 1st grade of elementary education, whereas the second study was carried out in the 1st and 2nd grades. All schools belong to an upper-middle sociocultural group. In the first study, three different teachers were in charge of the experimental groups, and in the second study only one teacher implemented the PEIM in both groups. In both cases, we studied the influence of the program in solving verbal problems. However, in the second work, with the aim of studying the development of students depending on their ability, we also used other complementary tests so as to check their mathematical competence and IQ.

## Materials and Methods

### Participants

#### Experiment 1

In order to empirically verify the PEIM’s effectiveness, we randomly chose five groups in 1st grade of elementary education in Madrid’s Public Schools, in upper middle-class residential areas. Two of these groups were used as control groups, whereas the three remaining classes, the experimental groups, followed the PEIM throughout the school year.

#### Experiment 2

The sample is made up of a group of 92 students from 1st and 2nd grade of elementary education in a charter school of Zaragoza. They were divided into four large groups of 23 children each. Two of them were from 1st grade and the other two were from 2nd grade: totaling 46 in 1st and 46 in 2nd grade. We established a control group and an experimental group in the 1st graders, as we also did in 2nd graders. At the beginning of the research, 1st graders had an age range between 5.9 and 6.8 years (*X* = 6.34), and 2nd graders belonged to the age range between 6.10 and 7.7 (*X* = 7.42). Along the study, three experimental deaths occurred, a fact that is not already considered for the sample participants.

### Stimuli

#### Experiment 1

Six of the simplest verbal problems fall into the four main categories according to the ranking established in the Bermejo et al. (1998). These problems were formulated in both their additive and subtractive forms, except in the latter case for combination problems. Likewise, numerical expressions of addition and subtraction were applied with the unknown quantity both in the result and in the second term. The teachers of the experimental groups passed three questionnaires. Questionnaire I was used to examine the knowledge that these teachers had on the specific development of the mathematical content in these students. Questionnaire II based on checking teachers’ beliefs and attitudes toward teaching-learning mathematics. And questionnaire III was used to look for information on self-evaluation about the impact that PEIM had had on their teaching. Eventually, we prepared an observation guide to register classroom dynamics.

#### Experiment 2

The students carried out several mathematical tests to evaluate their mathematical competence and a test to evaluate the groups’ homogeneity. The tests we used were the following:

(1) The BADyG E1 test to have an estimate of each participant’s general intelligence.
(2) For solving verbal problems, we presented the students a total of eighteen verbal problems, following Bermejo’s classification (1990).
(3) Tedi-Math to measure the mathematical competence to assess the different areas: counting, numbering, understanding the number system, doing operations and solving verbal problems.

### Procedure

#### Experiment 1

Firstly, we carried out an individual evaluation of all the students from both experimental and control groups to diagnose the previous mathematical knowledge and elaborate their mathematical profile. It consisted of an individual testing of the verbal problems and the numerical expressions mentioned above. The same tests were applied equally in the middle of the course to the experimental group (second evaluation), and at the end of the course they were applied to both control and experimental groups (third evaluation). All evaluations were recorded to facilitate a thoroughly detailed analysis. When the first evaluation was completed, questionnaire I was given to the teachers of the experimental group. Subsequently, all teachers also took questionnaire II. And finally, the teachers of the experimental group attended a 10 h seminar for several days, in which they were offered information about general child development and especially about specific mathematical development: addition, subtraction, verbal problems, strategies, errors, etc. In order to contextualize and specify all this information, we frequently offered them videos made by the same researchers to observe how different children solved the problems and the tasks proposed. At the end of the seminar, each teacher was provided with the “mathematical profile” of each of their students, made from the first evaluation we did at the beginning of the course. At the end of the course, questionnaire II was passed again to the teachers of the experimental groups to compare the results with those obtained in the first testing, as well as with the results obtained by the students in the groups in the last evaluation.

To evaluate the dynamics of the classroom, we maintained monthly meetings with the teachers of the experimental group and prepared an observation guide that included, among other things, the teachers’ interventions, the students’ initiatives, the type of activity of the students, etc. This record was carried out twice a month from February to April. Finally, we ended up passing questionnaire III for self-assessment on the impact that the PEIM had had on the teachers’ instructional activity of the experimental groups.

#### Experiment 2

We carried out the classic experimental design where two groups participated, one control group and one experimental group. The distribution of the subjects in the different groups was randomly carried out in a stratified way in order to have a similar number of boys and girls both in the experimental and in the control groups. The TEDI-MATH test for checking mathematical knowledge was given to all the groups individually before the educational intervention was performed, as well as the measurement of eighteen verbal problems to assess their resolution. The problems presented fall into five different types of addition problems: change, combination, comparison, equalization and referential, and four types of subtraction: change, comparison, equalization and referential. Each problem was presented according to two variables in terms of the place of the unknown, either at the beginning or at the end. The numbers used in the measurements were modified in accordance with the grades (1st and 2nd graders) to soften or increase the difficulty. The result that the students had to provide did not have to exceed numbers 10 and 20, respectively, since our interest was not in testing their ability in operating with larger numbers, but in the reasoning applied to the different situations. These measurements were repeated at the end of the intervention (post-test).

The BADyG E1 intelligence test was also applied to the entire sample in groups.

The educational intervention was carried out in the mathematics classes throughout the school year. In one of the classes the teacher applied the constructivist program PEIM, while the control group continued working with a traditional methodology, that is, using calculation procedures and the textbook. The most outstanding tasks in the implementation of the PEIM were the verbal problems close to their immediate surroundings, because these allowed introducing other mathematical concepts as well as reasoning activities. They were proposed through the use of ICTs (e.g., power point, Prezi, etc.), and students worked on expendable materials that we handed them out (e.g., stickers, multi-cubes, jellybeans, etc.). The procedure applied to the development of the activities responded to Bruner’s representation (1964, 1973). The problem was, in first place, proposed to work orally and avoid difficulties in reading and writing, which could condition problem resolution; and then, it was actively developed in the different groups through the manipulation of objects. Once the students had exchanged either their methods of resolution or their mathematical opinions about the concepts, they proposed other activities that were carried out in an iconic way, that is, making a graphic representation. The last step in concept forming was to translate it into mathematical language, that is, doing a symbolic representation. For all the activities, they were provided with the time to share their proposal with the rest of the class.

These activities were supplemented with supermarkets, bingos and sessions in which students invented their own problems based on the conditions provided, giving them time to reflect on their knowledge, manage relationships about the corresponding operations and become aware of the errors committed in the relationships established, so that they could correct them.

## Results

### Experiment 1

Bearing PEIM in mind, we first selected the main mathematical contents of addition and subtraction. In order to do so, the best way to teach these contents is to propose familiar and contextual verbal problems in difficulty order, as proposed in Bermejo et al. (1998). Additionally, we collected information on the use of numerical expressions.

In the first evaluation, the results obtained in the ANOVA show that the *Task* is the only significant factor [*F*(3, 285) = 3.62, *p* < 0.05], while the *Group* and *Operation Type* factors were not significant. In fact, the means of the groups and types of operation do not show important variations, while the verbal problems of change proved easier than those of comparison and equalization.

The second evaluation was carried out all along February with the aim of verifying only the experimental groups’ progress in mathematical learning. The results also showed significant differences in the *Task* factor, with significant differences between the following problems: change and equalization, compare and equalization, and equalization and numerical expressions problems. The *Group* factor is not statistically significant, although the mean of group III is usually higher than that of the other groups.

At the end of de course, we carried out the third evaluation to all participants, both experimental and control groups. The results show significant effects on the factors *Group* [*F*(4, 94) = 9.42, *p* < 0.01], and *Task* [*F*(3, 282) = 9.48, *p* < 0.01]. In fact, in this evaluation the means of the three experimental groups exceeded significantly the means obtained from the control groups (Gex I = 1.18; Gex II = 0.92; Gex III = 1.41; Gc IV = 0.73; Gc V = 0.39), which allows us to affirm, at least provisionally, that the application of the PEIM had a positive effect on the students of the experimental groups. On the other hand, although there are no significant differences between the scores obtained by the three experimental groups, it is clear that group III obtained the best results, followed by group I and then group II (see Table 1).

**TABLE 1:** Global means of all groups in three evaluations.

**Evaluations**	**G.I**	**G.II**	**G.III**	**G.IV**	**G.V**
1	0.55	O.47	0.67	0.56	0.42
2	0.96	0.73	0.99	–	–
3	1.28	0.97	1.51	0.85	0.52

As far as the teachers’ educational profile is concerned, questionnaire I showed that they had generally little knowledge of addition and subtraction verbal problems, a fact that limited themselves mainly to the change type of problems. This limited information was also shown when they were asked to judge the degree of difficulty of the different verbal problems, as well as the strategies used by children and their errors in each type of verbal problem, confirming the thesis that teachers used to evaluate their students focusing on the results rather than the processes used.

Questionnaire II focused on the teacher’s views and beliefs on the teaching-learning mathematics, specifically, what they know of constructivist principles, their application in the classroom and how evaluation is carried out. Among other results, we found that teacher 3 (Gex III) showed more systematic agreement on the constructivist perspective compared to other teachers, who were in greater disagreement when coming to the application of constructivist ideas in the classroom. In a second evaluation using the same questionnaire, teacher 3 (Gex III) confirmed the results obtained in the first evaluation, whereas teacher 1 (Gex I) showed a clear approximation to the constructivist principles.

The information obtained in questionnaire III suggests that the PEIM had a positive impact, in general, on the three teachers’ mathematics teaching to the experimental group. We can emphasize that teacher 1 found very importance the specific mathematical development of children and showed his interest in taking into account children’s strategies and errors when assessing and evaluating. Teacher 2 underscores, among other things, the importance of knowing and applying the different verbal problems of adding and subtracting in the classroom. And, finally, teacher 3 informs, among other things, of incorporating the different verbal problems into his teaching, as well as introducing changes in how to evaluate, by helping the child to reflect on the “mistakes made.” Summing up the results of questionnaire III, teacher 3 is the one who better knows and applies the constructivist approach in the classroom, followed by teacher 1 who shows special interest in the ideas of this approach and convinced of the instructional effectiveness it can provide.

The observation guide that we used to assess the constructivist dynamics in the classroom focused on four main areas: the teacher’s interventions and students’ initiative degree, the types of activities and the teaching resources and evaluation. With respect to the first area, teacher 3 allowed the student to discover the solution to the problem with some frequency, whereas the other two teachers preferred to explain themselves how to solve the problems. The three teachers marked the students’ assignments individually, and teacher 3 frequently explained individually the mistakes to the students. In general, the students of the three teachers solved the tasks individually, although with some frequency all the students taught by teacher 3 participated in the solution of the task. Concerning the types of activity, the algorithm was used by teachers 1 and 2 to work in general with addition and subtraction, although in most of the cases, they also proposed to work on change and combination problems. In contrast, teacher 3 used word problems to teach addition and subtraction, asking them to solve word problems, or formulate word problems extracted from some data. Regarding teaching resources to solve the problems (materials, drawings, etc.), teacher 3 was the only one to use some with his students. Finally, learning assessment was not carried out only from the students’ results, but rather all teachers also chose to evaluate the processes quite frequently.

### Experiment 2

The four groups were very similar in terms of intelligence and previous mathematical knowledge according to the BADyG E1 General Intelligence Test and TEDI-MATH tests. The differences between the control and experimental group regarding the difference in the scores obtained in the TEDI-MATH pretest and post-test applied to the students, were analyzed in an ANOVA 2 (groups: experimental, control) × 2 (time of measurement: pre-test, post-test), with a second intrasubject factor, since both groups had samples with a normalized distribution. The results showed a significance of the main effects, *F* = 282.95, *p* < 0.0000, and *F* = 113.73, *p* < 0.0000, for measurement time and group, respectively.

For the present work, we also found interesting the difference in scores obtained in the pre-test and post-test with respect to problem solving, which is also significant both in 1st and 2nd grade groups. As we did not obtained a normalized distribution in any of the courses with respect to the score difference variables, in 1st (*K*-Scontrol = 0.123; *p* = 0.200) and (*K*-Sexperimental = 0.215; *p* = 0.007) and in 2nd (*K*-Scontrol = 0.280; *p* = 0.000) and (*K*-Sexperimental = 131; *p* = 0.200), we needed to carry out a Mann-Whitney test on the difference in the correct score between the different moments, UMann Whitney = −5,226; *p* = 0.000 and UMann Whitney = −5.827; *p* = 0.000, respectively.

To carry out the differential study regarding the evolution of the learning process after the educational intervention, the analysis is performed with the sample that belongs to the experimental group, since we are interested in knowing if there is any correlation between the methodology used, the mathematical competence and the students’ ability. The analysis was carried out differentiating the course the students belonged to. We built a categorization of the students in the experimental group with respect to the IQ obtained in the BADyG E1 test, and then we analyzed the difference in the score obtained in solving problems with respect to the pre-test and post-test.

In order to accomplish this, we established three categories according to the IQ of the students following the classification of the Wechsler scale, which has an average equal to 100 and a standard deviation equal to 15, implying that the values relate with the categories of most common diagnostic use corresponding to low ≤ 89, 90 ≤ medium ≤ 110, and high ≥ 110.

**FIGURE 1:**
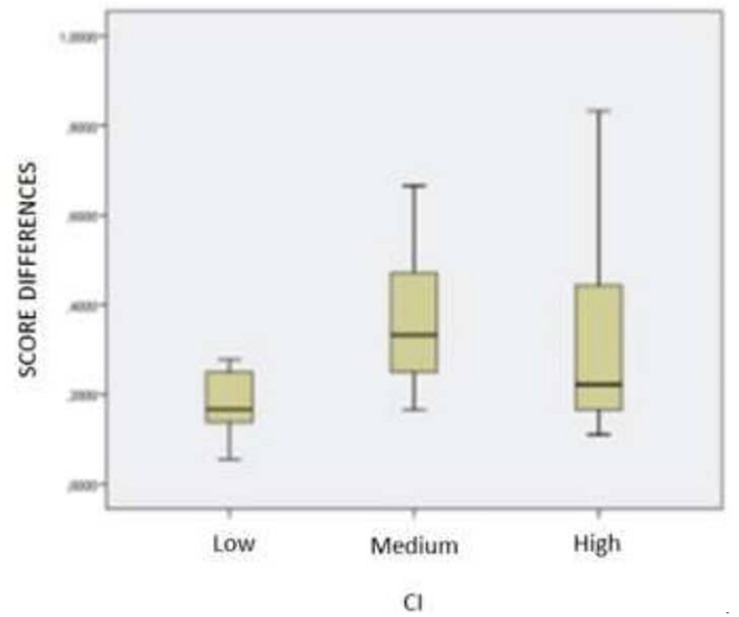
1st grade group, evolution of learning with respect to IQ.

In 1st graders’ sampling, the hypothesis of sample normality is accepted, so an ANOVA, *F* = 2,324 and *p* < 0.124, is carried out, which makes it acceptable that the evolution of learning is similar in all categories, what is graphically reflected in the box of boxes and mustaches (see Figure 1).

**FIGURE 2:**
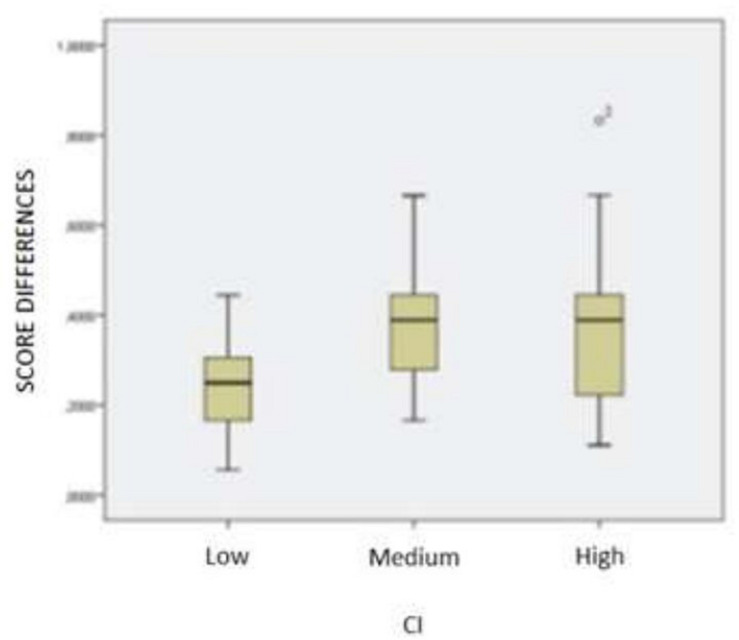
2nd grade group, evolution of learning with respect to IQ.

Likewise, in 2nd grade, the same categorization is established whereby Shapiro-Wilk allows accepting the normality of the samples in each category, applying the ANOVA analysis, *F* = 0.632, *p* < 0.542, that is, the results show a similar learning evolution in all three categories, which we can see graphically in Figure 2.

## Discussion

**FIGURE 3:**
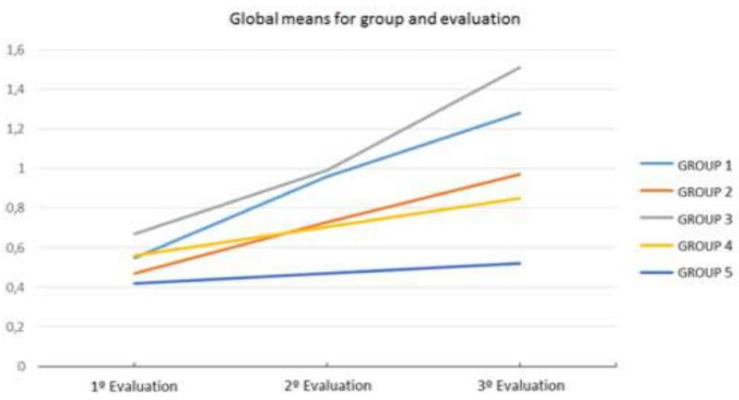
Global means for group and evaluation.

Teaching and instruction are two closely related concepts in educational practice. A modification in teaching by implementing a more constructivist instruction, which starts in knowing the student’s specific mathematical development, seems to have positive effects on the development of the mathematical competence. It has been shown that individuals who have received knowledge passively tend to continue using mathematics in this way both in their jobs and in their daily life (Boaler and Selling, 2017). In experiment I it can be stated that there exists a relationship between the level of application of constructivism in the classroom and the mathematical performance of children, as can be seen in Figure 3. This is the case of teacher 3, who knows and applies constructivist principles better in the classroom, and whose students achieve higher scores in mathematics. Likewise, we find clear differences concerning evaluation, in the sense that even if, before starting the implementation of the PEIM the teachers focused their evaluation on the results obtained, they eventually analyzed and evaluated the processes as well.

The results of the second experiment are highly consistent with what can be found in the first experiment. The differences between the experimental groups, in which a constructivist methodology was developed, and the control group are significant both in their mathematical competence and in solving problems. Furthermore, the strategies used in the experimental group bring about developmental differences compared to the control group.

If we look at the students’ learning evolution in terms of their capability, the data obtained shows that their performance may be slightly higher in students with more abilities; however, it is not the case of their learning evolution. This is especially important since the students who show more difficulties in the area of mathematics, have a similar evolution to the rest of the students. We think that, for those students, the program could be an effective alternative. At a qualitative level, it was possible to observe that in the experimental group, the students who did not have strategies to approach problem solving, due to some initial blocked state, they started to be more self-confidence and achieved important results, because once they had addressed the problem and could not solve it, they tried to solve it in many other ways. If math classrooms do not actively engage students, by giving positive messages and opportunities to all students (Boaler and Sengupta-Irving, 2016; Boaler, 2015; Boaler and Selling, 2017), this may be due to some mismatching between the math learnt in school and the math they need for today’s adaptative innovative and technological world.

One of the reasons of PEIM’S positive results is that it allows students in the experimental group to carry out intellectual work, since, through discovery, they were able to develop and reason methods to solve problems in the context of the real world and based on their prior knowledge. However, the control group was guided toward resolution procedures that they could understand and apply but did not actually internalize.

The constructivist methodology encourages students to confront and agree on ideas, besides allowing them to structure knowledge from their own cognitive processes, integrating their own ideas based on those learned with their classmates. The benefits of peer work have been demonstrated in meta-analysis studies in which they conclude that the benefits in elementary education outnumber those in secondary education (Alegre et al., 2019a; Anderson et al., 2005). In the experimental group, a certain critical attitude in problem solving was detected, leaving aside more procedural mechanisms that they had acquired in previous years. The approach was to understand the situation raised rather than choosing an algorithm that would allow them to resolve it.

Contextualizing the tasks and having been posed in different ways allowed students with more difficulties to have a starting point for solving them, that is, it allowed students to accommodate and assimilate those tasks with respect to experiential situations they had experienced before. This also allowed them to build resolution models and a generation of tasks in a more affordable way. This is the reason why their significantly improved performance contrasted with other groups.

Considering that the classroom climate of the experimental group brought to light working in a cooperative learning context, it is not surprising that its evolution was similar taking into consideration the clearly positive effects that these groups generally have on learning. There are relatively few environments in which students actively interact with mathematics and participate in a wide range of practices (Jacobs et al., 2006; Litke, 2015; Boaler and Selling, 2017).

## General Considerations

The classes in which we worked through the PEIM allowed for a series of favorable conditions for the improvement of learning to occur, such as participation, dialogue, construction of knowledge, and the ability to reason and debate. In addition, the development of group work contributed to improve the efficiency and persistence in searching for solutions. The teacher will guide the student’s knowledge construction process and will be the one who manages an adequate classroom dynamic.

The positive effects of the PEIM are evident, both in the changes that occurred in the improvement of the mathematical performance, contributing to the students’ search for solutions and strategies as well as in the classroom dynamic. In the most traditional teaching processes, the informal knowledge with which the student arrives in school is not taken into account, ranging from teaching arithmetic operations to verbal problems, which produces a kind of schizophrenia in children due to the lack of a continuum from the daily life mathematics to the school mathematics. Furthermore, the student learns arithmetic independently of verbal problems, a fact that leads the student to make a mechanical choice between two algorithms when finding a solution to verbal problems (Harris and Graham, 1994).

Many students do not address verbal problems by building a mental representation of the problem, from which to apply the relevant strategies to solve it. In contrast, what they usually do is to follow the procedure of finding key words in the verbal formulation that may give a false clue about the type of operation they should choose. For this reason, it is convenient for students to be accustomed to deal with verbal problems by first seeking the construction of its mental representation. By searching for keywords, it will not only condition their success at mandatory educational levels such as elementary education, but also all along their school years.

For this reason, it would be advisable for teachers to start working with students’ prior knowledge, the same way as Luis Vives had kindly defended in the sixteenth century, and a century later, Jean Jacques Rousseau put forward in the prolog of his hallmark educational treaty, *Emilio*: “Start by studying to your students, because surely you don’t know them.” The effectiveness of these classical authors and their teaching is highly significant when present-day teachers know extensively about the specific development of each and every fundamental school mathematics content. Therefore, bearing in mind such influential views and knowledge, we can guide, collaborate and effectively help the child to walk along “step by step” into acquiring the specific mathematical content.

Children’s manipulating materials constitutes an indispensable element to streamline their mathematical learning. In this respect, notorious neuroscience authors such as Dehaene (1997) and Butterworth (1999) confirm this fact when they advise instructing mathematics by intuitively reasoning and manipulating materials. Rivera-Rivera (2019) adds that “the manipulation of materials generates a brain activity that facilitates understanding. If what is being learned is understood and comprehended, various brain areas are activated, meanwhile if it is memorized without sense, neuronal activity is much poorer” (p. 166). Along these lines, Bermejo and Lago (1988) clarify and empirically show that the use of materials is very positive at the beginning of the mathematical content learning, although it can be an obstacle once this phase has been overcome. Likewise, some authors defend the importance of the use of fingers in mathematical development (Barrocas et al., 2020; Fischer et al., 2020). Therefore, it is convenient to “de-algorithmize” the mathematics classes since the algorithm is only an instrument for solving tasks, and not their ground foundation. In addition, we must insist on presenting the tasks considering their degree of difficulty, analyzing the semantic structure and the place of the unknown, which condition their difficulty. Non-routine problems are an opportunity that allows the child to find a solution in an alternative way, using his divergent thinking.

Moreover, although the PEIM has been applied in this work to 1st and 2nd grade elementary school students, we believe that the PEIM can also be applied to other educational levels. We should highlight that one of the limitations that we can find is to develop this program in very large groups since the PEIM advocates for the personalization of mathematics teaching, tailored to each student’s mathematical profile. It would be a matter of choosing the appropriate mathematical content according to the developmental-mathematical level of the participants, and having teachers received the corresponding training. If the students’ educational levels are higher, the use of materials in teaching could be less significant, or in any case, it would be necessary to choose the appropriate ones (Bermejo, 2014). Neither do we have data on students with learning difficulties. However, we think that the PEIM could be applied with positive results to these schoolers. It would consist, on one hand, in teachers selecting the appropriate mathematical contents according to the participants’ mathematical knowledge; and on the other, in teachers receiving the appropriate training to teach these students the mathematical contents, considering also, their personal characteristics. In this case, it would be necessary to know the peculiarity of the students so that the classroom dynamic can based on constructivism.

## Conclusion

To summarize, we would like to conclude by providing the basic ideas that make up a teacher’s training program:

- The main constructivist foundations of the PEIM: (a) children will construct their own knowledge; (b) instruction guides and supports their knowledge construction; (c) instruction will focus on understanding and solving problems; and (d) specific mathematical development will form the foundation for sequencing instructional objectives.
- Profile of the constructivist student: (a) he constructs his own knowledge; (b) is mentally and manually active; (c) acquires significant knowledge; and (d) is autonomous and independent in constructing their knowledge.
- Profile of the constructivist teacher: (a) the child constructing his knowledge is the protagonist of the classroom; (b) learning mathematics involves understanding the procedures and solving problems; (c) schoolchildren come with previous knowledge before class instruction; (d) the teacher has an active attitude, by listening and asking his students, continuously evaluating all the processes and intervening whenever appropriate; and (e) personalized interaction with students.

## Data Availability Statement

The raw data supporting the conclusions of this article will be made available by the authors, without undue reservation.

## Ethics Statement

Ethical review and approval was not required for the study on human participants in accordance with the local legislation and institutional requirements. Written informed consent to participate in this study was provided by the participants’ legal guardian/next of kin.

## Author Contributions

VB: team coordination, theoretical framework, data analysis experiment, and intervention program. PE: data analysis experiment, intervention program, results, and discussion. IM: editing coordination, revision, final proofreading, and discussion. All authors contributed to the article and approved the submitted version.

## Conflict of Interest

The authors declare that the research was conducted in the absence of any commercial or financial relationships that could be construed as a potential conflict of interest.
